# Supplementary material for: The Crosstalk between Cardiac Lipotoxicity and Mitochondrial Oxidative Stress in the Cardiac Alterations in Diet-Induced Obesity in Rats
Source: Cells. 2020 Feb 17;9(2):451. doi: 10.3390/cells9020451 (PMC7072852; doi:10.3390/cells9020451)
Supplement: Supplementary file 1 [file cells-09-00451-s001.pdf]

Supplemental figure legends

Figure S1

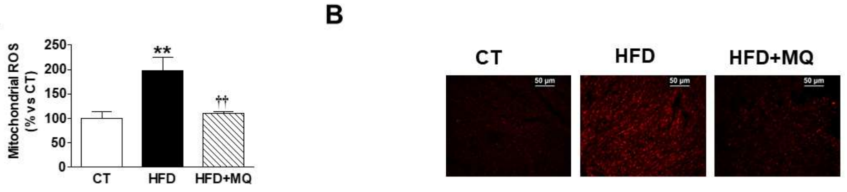

Figure 1. Effects of the mitochondrial antioxidant MitoQ (MQ) on mitochondrial superoxide anion levels. Hearts from rats fed a standard diet (CT) or a high fat diet (HFD) treated with vehicle or with MQ, the mitochondrial antioxidant (200 μM), were analyzed. (**A**) Quantification of mitochondrial cardiac levels of superoxide anion and (**B**) Representative microphotographs of cardiac sections labeled with MitoSox are presented. Magnification (x40). Bar graphs represent the mean± SEM of 5 animals. **p<0.01 *vs*. control group. ††p<0.01 vs HFD group.

Figure S2

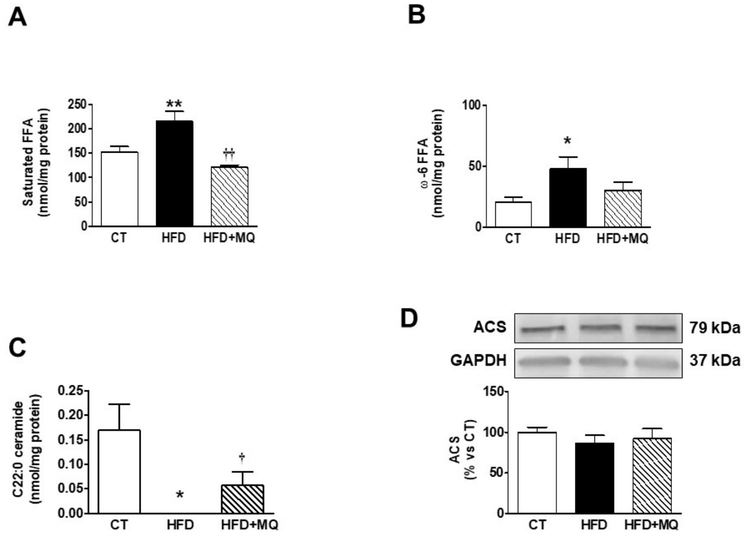

Figure 2. Impact of the mitochondrial antioxidant MiToQ on mitochondrial lipid species and protein levels in heart of obese rats. Hearts from rats fed a standard diet (CT) or a high fat diet (HFD) treated with vehicle or with MQ, the mitochondrial antioxidant (200 μM), were analyzed. (A) Mitochondrial free fatty acids (FFA) enriched with saturated acids, (B) Mitochondrial FFA enriched with ω-6 acids, (C) Mitochondrial ceramides enriched with (C22:0) and (D) Protein expression of acetyl CoA synthetase (ACS) are presented. Bar graphs represent the mean±SEM. of 6-8 animals normalized to glyceraldehyde-3-phosphate dehydrogenase (GAPDH).*p<0.05; **p< 0.01; vs control group. †p<0.05 ††p<0.01 vs HFD group.

Figure S3

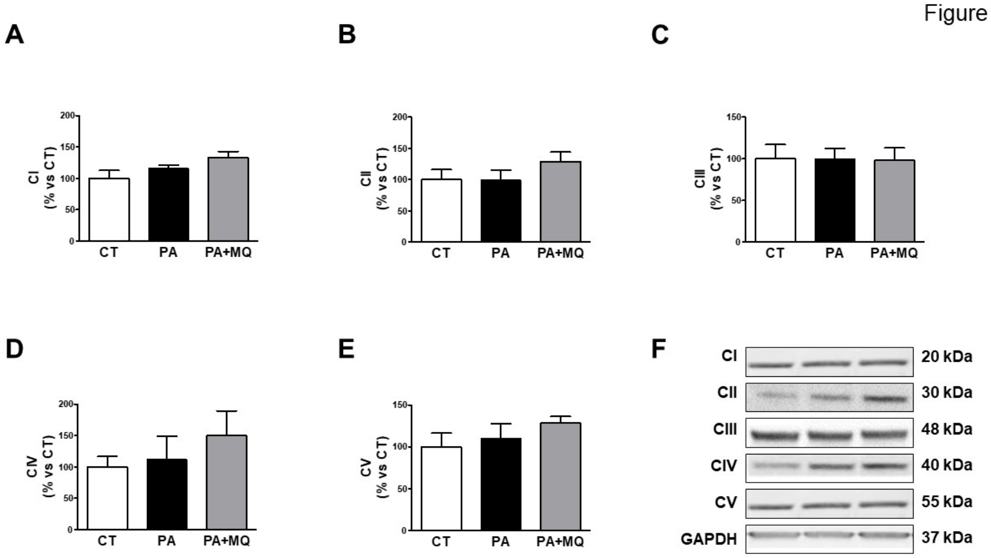

Figure 3. Effects of antioxidant MiToQ on complexes protein levels in palmitic-acid-treated H9c2 cells. Protein expression of mitochondrial complexes (**A**) I (subunit NDUFB8), (**B**) II (SDHB subunit of complex II) (**C**) III (core protein 2 UQCRC2), (**D**) IV (MTCO1 subunit 1)and (**E**) V (alpha subunit) in cardiac myoblasts treated for 24 h with palmitic acid (200 μM) in the presence or absence of the mitochondrial antioxidant MitoQ (MQ; 5 nM). (**F**) Representative blots for mitochondrial complex protein expressions. Bar graphs represent the mean ± SEM. of 6-8 animals normalized to glyceraldehyde-3-phosphate dehydrogenase (GAPDH).
